# Sex-specific response to A1BG loss results in female dilated cardiomyopathy

**DOI:** 10.1186/s13293-025-00713-8

**Published:** 2025-04-23

**Authors:** James I. Emerson, Wei Shi, Frank L. Conlon

**Affiliations:** 1 Departments of Biology and Genetics, University of North Carolina at Chapel Hill, Chapel Hill, NC USA; 2 McAllister Heart Institute, University of North Carolina at Chapel Hill, Chapel Hill, NC USA; 3 Mary & Dick Holland Regenerative Medicine Program, University of Nebraska Medical Center, Omaha, USA

**Keywords:** A1BG, Sex differences, Heart, Cardiac, DCM, Conduction, Intercalated discs

## Abstract

**Background:**

Cardiac disease often manifests with sex-specific differences in frequency, pathology, and progression. However, the molecular mechanisms underlying these differences remain incompletely understood. The glycoprotein A1BG has emerged as a female-specific regulator of cardiac structure and integrity, yet its precise role in the female heart is not well characterized.

**Methods:**

To investigate the sex-specific role of A1BG in the heart, we generated both a conditional A1bg knockout allele and an A1bg Rosa26 knockin allele. We employed histological analysis, electrocardiography, RNA sequencing (RNA-seq), transmission electron microscopy (TEM), western blotting, mass spectrometry, and immunohistochemistry to assess structural, functional, and molecular phenotypes.

**Results:**

Loss of A1BG in cardiomyocytes leads to persistent structural remodeling in female, but not male, hearts. Despite preserved systolic function in female A1bg^CM/CM^ mice left ventricular dilation and wall thinning are evident and sustained over time, consistent with early-stage dilated cardiomyopathy (DCM). Transcriptomic analyses reveal that A1BG regulates key metabolic pathways in females, including glucose-6-phosphate and acetyl-CoA metabolism. TEM imaging highlights sex-specific disruption of intercalated disc architecture in female cardiomyocytes. These findings suggest that the absence of A1BG initiates chronic pathological remodeling in female hearts, potentially predisposing them to DCM under stress or aging.

**Conclusion:**

A1BG is essential for maintaining ventricular structural integrity in female, but not male, hearts, leading to a chronic remodeling consistent with early-stage DCM.

**Supplementary Information:**

The online version contains supplementary material available at 10.1186/s13293-025-00713-8.

## Highlights

Female mice but not male mice with a cardiac muscle-specific conditional loss-of-function mutation in A1BG exhibit significant cardiac dysfunction indicative of dilated cardiomyopathy.

Female and male cardiomyocyte A1BG null hearts showed different gene expression patterns, indicating that the cardiac metabolic adaptations required in the absence of A1BG are sex specific.

Functional network analyses of the cardiac interactomes of female and male A1BG reveal that A1BG interacts with different sets of proteins based on sex, suggesting that these interactions are crucial for the sex-specific roles of A1BG in cardiac function and dilated cardiomyopathy.

**Supplementary Information:**

The online version contains supplementary material available at 10.1186/s13293-025-00713-8.

## Abstract (de)

Female mice with a mutation in the A1BG gene experience significant heart problems, such as an enlarged left ventricle and thinner heart walls, changes not observed in male mice with the same mutation. Female hearts exhibit different gene activity related to heart function and metabolism than males. The mutation impacts the structure of heart cell connections in females, resulting in impaired heart function and electrical activity, including longer times for the heart’s electrical signals to travel. These defects are similar to those observed in patients with dilated cardiomyopathy. The results indicate that A1BG plays a crucial role in how the female heart forms connections between cells and maintains its structure. The sex-specific requirements for A1BG in the heart could be attributed to the fact that A1BG interacts with different protein partners in male and female hearts. This research highlights the importance of A1BG in maintaining healthy heart function and structure in female mice, suggesting that targeting A1BG could help treat heart disease, especially in women.

## Introduction

Various human diseases exhibit significant sex differences in prevalence, treatment, and survival rates, including cancer, cardiovascular disease, autoimmune disorders, obesity, and chronic kidney disease [1–7]. In the context of cardiac health, these sex disparities are particularly notable [4, 6–18]. Basic physiological differences, such as higher resting heart rates, longer ventricular repolarization, and shorter atrial and ventricular conduction times in females, predispose them to distinct types of heart disease compared to males [4, 6, 8–12, 15, 16, 19, 20]. Understanding the cellular and molecular mechanisms underpinning these sex disparities in cardiac physiology and disease is crucial for improving patient outcomes and ensuring equitable care.

Dilated cardiomyopathy (DCM) is a condition marked by the enlargement and weakening of the left ventricle, leading to diminished cardiac output and potential heart failure [21–23]. This impairment disrupts the heart’s ability to circulate blood efficiently, resulting in complications such as arrhythmias and potentially sudden cardiac death [21–23]. DCM is the most common reason for heart transplantation in the U.S. and remains a leading cause of morbidity and mortality [24, 25]. Approximately half of DCM cases are genetically determined, while the etiology of the remaining cases is often unknown [26, 27]. Although the symptoms of DCM, including heart failure and arrhythmias, are similar in both sexes, women are typically diagnosed at an older age, potentially due to the protective effects of estrogen [28–32].

Sex-specific requirements in cardiomyocytes (CMs) may explain the differences in heart health between males and females. A recent study highlighted the role of Alpha-1-ß-glycoprotein (A1BG) as a key factor in this sex-specific cardiac function [33]. Female mice with a homozygous loss-of-function mutation in the A1BG gene exhibited severe cardiac dysfunction, including thinning of the left ventricular posterior wall and dilated left ventricles. In contrast, male mice with the same genetic modification did not show these abnormalities [33].

Our findings demonstrate that the loss of A1BG in female mice has profound sex-differential effects on cardiac function. Female mice with a homozygous loss-of-function A1bg gene mutation exhibited significant cardiac issues such as left ventricular dilation and decreased wall thickness. Transcriptomic profiling indicated that A1BG in female hearts influences the expression of genes related to glucose-6-phosphate and Acetyl-CoA metabolism and DCM, emphasizing the critical role of A1BG in maintaining cardiac homeostasis. In contrast, male mice did not exhibit the same phenotypic changes, affirming a sex-specific requirement for A1BG in heart function. Moreover, female but not male mice displayed altered intercalated disc structures and prolonged PR intervals on electrocardiograms, indicating impaired atrial conduction, all indicative of DCM. Further analysis revealed that A1BG interacts with sex-distinct sets of CM proteins, suggesting that these interactions underlie the sex-specific requirements for A1BG in cardiac function and DCM. These findings underscore the critical requirement of A1BG in women’s cardiac health and suggest potential pathways through which A1BG may contribute to sex differences in heart disease.

## Materials and methods

### Animal models

#### Generation of the A1bg conditional allele

*R26R-A1bg-3HA* and *A1bg flox* mice were generated using CRISPR/Cas9 technology at the Animal Models Core of UNC-CH. The Tnnt2-Cre line was purchased from the Jackson Laboratory (ID: 024240). Genotyping was performed using the following primers to confirm the presence of transgenic alleles. Tnnt2-Cre (F- 5’ TTGTTCCTTTAGCCCTGTGC 3’, R- 5’ AGGCAAATTTTGGTGTACGG 3’) *R26R-A1bg-3HA*(F1-5’ATGTCTCTGCTGGCTACTGTACTG3’,F2-5’GTGAATGGGCCACCACCCAAG3’,R-5’GGATAGGATCCTGCATAGTCCGG 3’ ) *A1bg flox* (F- 5’ GTGTTCTTGGGAAGGGTTCA 3’, R- 5’ CAGCCAGAACCCTTAGTGTAGT 3’). Mice were sacrificed between 4 and 8 weeks for all experiments, and hearts were perfused with 1xPBS and dissected for use in proteomics and immunopurification analysis or perfused with 4% paraformaldehyde (PFA)/ 0.1% Tween-20/ PBS for immunohistochemistry and H&E analysis or dissected and homogenized in Trizol for RNA extraction. Mice were housed at a controlled temperature of 25 ± 1˚C, with a 12-h light/12-h dark cycle, with lights on from 07:00–19:00. Standard rodent chow and water were provided throughout the study. The Institutional Animal Care and Use Committee of UNC-CH approved this research (21 − 006, 22–257), which adhered to the Guide for the Care and Use of Laboratory Animals.

#### Generation of the A1bg ROSA26 Knock-in allele

A Cas9 guide RNA targeting the mouse Rosa26 1st intron (protospacer sequence 5’- GGAGTTGCAGATCACGA − 3’) was cloned into a T7 promoter vector. The vector was subjected to T7 in vitro transcription. The product was purified with a spin column containing microinjection buffer (5 mM Tris-HCl pH 7.5, 0.1 mM EDTA). A donor plasmid was generated to target ES cells. The donor included Rosa26 gene homology arms flanking a neomycin resistance cassette, CAG promoter, LoxP-STOP-LoxP element, mouse A1bg cDNA with c-terminal 3xHA tag, Woodchuck Hepatitis Virus Posttranscriptional Regulatory Element (WPRE), and rabbit beta-globin polyadenylation sequence. The donor vector was prepared by Qiagen High-Speed Maxiprep protocol and resuspended in a microinjection buffer. Recombinant Cas9 protein was expressed in E. coli and purified by the UNC Protein Expression and Purification Core Facility. ES cell line C57BL/6 N-PRX-B6N #1 was nucleofected with 1 µM Cas9 protein, 1.2 µM guide RNA, and 20 ng/µl supercoiled donor vector. Clones were selected with G418, and positive clones were identified by PCR screening. Positive clones were verified by Southern blot, and two clones were microinjected into Albino-C57BL/6 blastocysts for chimera formation. Chimeras were mated to Albino-C57BL/6 N females for germline transmission of the targeted allele. ES cell clone E2 gave germline transmission of the targeted allele. Heterozygous F1 animals (R26R-A1BG-3HA+/-) were bred to wild-type mice, and the genotypes were confirmed by sequencing and PCR. To generate a cardiac conditional allele, R26R-A1BG-3HA+/- mice were crossed with the cardiomyocyte-specific Tnnt2Cre/+ [34] to generate Tnnt2Cre/+; R26R-A1BG-3HA+/-; these mice were intercrossed to generate homozygous Tnnt2Cre/+; R26R-A1BG-3HA+/+ (A1BG KI) mice.

#### Histological analysis

Hematoxylin-eosin staining was performed as described [35] with *A1bg A1BGCM/CM* and control mice at 4 weeks of age (4 males and 4 females for each genotype). Histology sections were imaged using a BX61 brightfield microscope at 20X magnification. ImageJ was used for tile stitching and subsequent analysis. To determine ventricular wall thickness by ImageJ, pixel size was normalized to µm, and measurements were averaged over three fields for individual hearts.

#### Immunohistochemical analysis

Hearts from *n* = 2 male and female *A1bg KI* and control mice (4-weeks old) were fixed in 4% PFA/ 0.1% Tween-20/ PBS at 4˚C overnight (o/n), then dehydrated by sucrose gradient (15% o/n then 30% o/n) before embedding in OCT and cryosectioning. Immunofluorescent staining was performed with antigen retrieval on 10 μm coronal sections as described [35]. Sections were co-stained with primary antibodies rabbit anti-HA (CST37245, 1:400; Cell Signaling Technology), mouse anti-tropomyosin (CH1, 1:50; Developmental Studies Hybridoma Bank), and rabbit anti-A1BG (Ab231805, 1:250; Abcam). Secondary antibodies used were Alexa 546-goat-anti-rabbit (1:500; Molecular Probes) and Alexa488-goat-anti-mouse (1:500; Molecular Probes). Images were acquired using a Zeiss LSM 700 laser scanning confocal microscope, and ImageJ was used for analysis.

#### Transmission Electron microscopy

Animals (8 weeks old) were perfused with a fixative containing 2% paraformaldehyde and 2.5% glutaraldehyde in 0.15 M sodium phosphate buffer, pH 7.4. After perfusion, the tissues were removed and cut into ~ 2 mm strips and stored at 4 degrees Celsius in the fixative before processing for electron microscopy. Following three rinses with 0.15 M sodium phosphate buffer, the samples were post-fixed at ambient temperature for 1 h in 1% osmium tetroxide in sodium phosphate buffer [36]. The tissues were rinsed in deionized water and dehydrated with increasing concentrations of ethanol (30%, 50%, 75%, 100%, 100%, 100%, 15 min each) and put through two changes of propylene oxide (15 min each). Tissue samples were infiltrated with a 1:1 mixture of propylene oxide: Polybed 812 epoxy resin (1 A:2B formulation, Polysciences, Inc., Warrington, PA) for 3 h, followed by a 1:2 mixture of propylene oxide: Polybed 812 epoxy resin for 6 h, and then infiltrated with 100% resin overnight. The tissue pieces were embedded in fresh epoxy resin and polymerized for 24 h at 60˚C. Using a diamond knife, 1-µm semi-thin sections were cut, mounted on slides, and stained with 1% toluidine blue to examine by light microscopy and isolate the region of interest. Ultrathin Sects. (70–80 nm) were cut with a diamond knife and mounted on 200 mesh copper grids, followed by staining with 4% aqueous uranyl acetate for 12 min and Reynold’s lead citrate for 8 min. Samples were observed with a JEOL JEM-1230 transmission electron microscope operating at 80 kV (JEOL USA, Inc., Peabody, MA), and images were acquired with a Gatan Orius SC1000 CCD Digital Camera and Gatan Microscopy Suite 3.0 software (Gatan, Inc., Pleasanton, CA) [37].

#### Electrocardiogram and echocardiogram analysis

EKG analysis of 4 to 6-week-old *A1BG*^*CM/CM*^ and control mice (*n* ≥ 10 males and 10 females for each genotype) was performed as previously described [38]. EKGs were performed by live restraint of non-anesthetized mice. EKGs were analyzed using the Vevo labs application, whereby at least ten consecutive waveforms were averaged to obtain EKG parameters for each mouse. Echocardiogram analysis was performed on *n* = 11 mice aged 4 to 6 weeks and *n* = 7 mice aged 6 months, as previously described [39]. All EKG and echocardiogram analyses were performed by trained technicians blinded to mouse genotype.

#### Immunopurification coupled with Mass-spectrometry

Two male and two female hearts of each genotype (*A1bg KI* and control) (mice were 4 weeks old) were pooled for immunopurification, performed as described with minor alterations [40]. Taking advantage of the C-terminal 3x HA tag on A1BG in KI mice, we used anti-HA magnetic beads (Pierce 88837) to immunopurify A1BG protein complexes. *R26R-A1BG-3HA* mice without Cre recombinase were used as controls. Immunopurification was performed as lysates were rocked for 1 h at 4˚C with beads. The complex was eluted after six washing steps with 1xNuPAGE LDS sample buffer (Thermofisher NP0007)/5% 2-mercaptoethanol (BME) for 10 min at 95˚C. The immuno-isolated proteins were resolved (~ 1 cm) by SDS-PAGE on a NuPAGE 4–12% Bis-Tris Gel (ThermoFisher) and visualized with Coomassie blue. Samples were submitted to the UNC Hooker Proteomics Core, where they were subjected to in-gel trypsin digestion as reported [40, 41]. Peptides were analyzed using scaffold version 5. The peptide threshold was set to 95%, the minimum number of peptides set to 2, and the protein threshold set to 99%. Samples enriched by HA immunopurification compared to control were filtered by having at least a 1.5-fold change greater number of peptides than the number captured in control samples. Proteins relating to ubiquitous cellular processes were also removed. Differentially enriched proteins in males and females were determined by at least a 1.5-fold difference between male and female IP samples.

#### Transcriptome analysis

Transcriptome analysis (RNA-sequencing) was performed as previously described [39]. Briefly, heart tissues of 4-week-old male and female A1bg KO and control mice were perfused in cold PBS and were harvested. The whole hearts were homogenized in Trizol reagent and the RNA was isolated using the PureLink RNA Mini Kit (ThermoFisher). Purified RNA was subjected to two rounds of oligo-dT selection and converted into cDNA to generate RNA-seq libraries. Libraries were sequenced (150-bp paired-end reads; Illumina HiSeq 2500) to a target depth of > 3 million reads. Using STAR via the bcbio-nextgen RNA sequencing pipeline, reads were aligned to the mm10 reference genome. DESeq2 (DESeq2_v1.18.1) in R (v3.4.3) was used to perform RNA-seq analysis. R scripts used to analyze this data are available upon request. Genes > 0.5 log2 fold change and adjusted p-value < 0.05 were deemed significant.

### Statistical analysis

Statistical analysis was performed using Prism9 software. ANOVA with Tukey’s test was used to determine significance between three or more groups. Mann-Whitney test was used to determine the significance between the two conditions.

## Results

### Loss of A1BG and Sex-Differential cardiac effects on the left ventricle

**Fig. 1:**
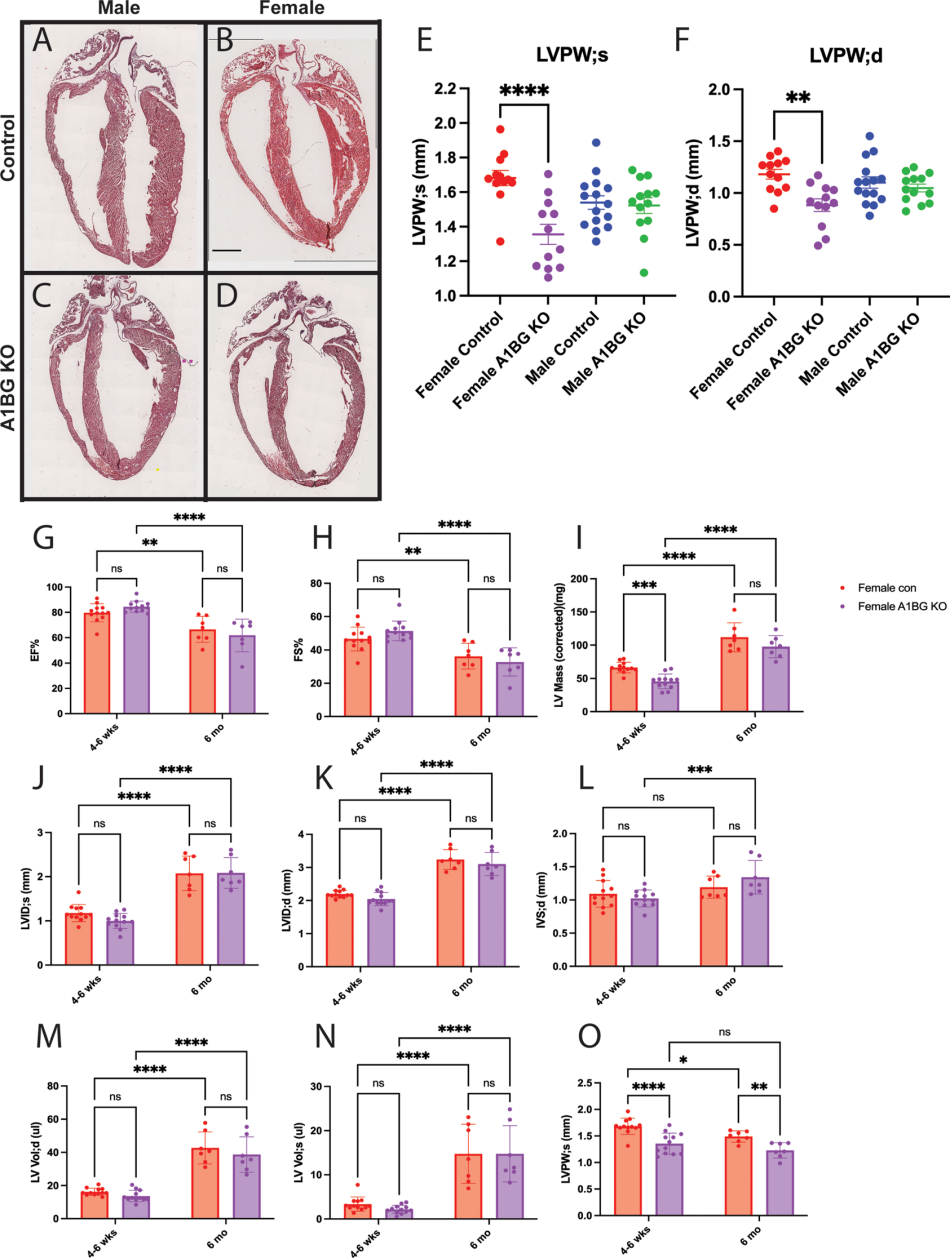
Loss of A1BG and Its Sex-Differential Cardiac Effects on the Left Ventricle. (**A**) Representative images of (**A**) male control, (**B**) female control, (**C**) male A1bg^CM/CM^ (A1BG KO), and (**D**) female A1bg^CM/CM^ (A1BG KO) hearts stained with hematoxylin and eosin. (**E**) Echocardiogram data (*n* = 11 mice of each genotype) for left ventricular posterior wall thickness in (**E**) systole (LVPW; s) and (**F**) diastole (LVPW; d). (**G**) Graph comparing female control and female A1BG KO mice at 4–6 week timepoint and 6 month for (**G**) ejection fraction (EF), (**H**) fractional shortening (FS), (**I**) left ventricular (LV) mass, (**J**) LV interior diameter in systole, and (**K**) diastole, (**L**) interventricular septum thickness, (**M**) LV volume in systole, and (**N**) diastole, and (**O**) LV posterior wall thickness in systole. Significance was determined by ANOVA with Tukey’s post-hoc test. **** indicates *p* < 0.0001, *** indicates *p* < 0.001, ** indicates *p* < 0.01, * indicates *p* < 0.05

Female mice homozygous for a cardiac muscle-specific conditional loss-of-function mutation in A1BG (A1bg^CM/CM^) exhibit structural remodeling of the left ventricle, characterized by an enlarged ventricular chamber and compromised interaction with the intraventricular septum [33]. Histological examination of 4- to 6-week-old mice validated and extended these findings, revealing ventricular dilation specifically in female mutants, a condition absent in their male counterparts (Fig. 1A-D). Echocardiographic analyses confirmed this observation (Fig. 1E, F) and further demonstrated that female, but not male, A1bg^CM/CM^ mice exhibited a significant reduction in left ventricular posterior wall thickness during both diastole and systole (Fig. S1A). In contrast, we find that male A1bg^CM/CM^ mice demonstrated a decrease in left ventricular volume and interior diameter during both diastole and systole while both sexes showed a decrease in left ventricular mass (Fig.S1B). The findings indicate that A1BG plays a critical role in maintaining the integrity of the ventricular wall in female hearts. Its absence leads to adaptations in the heart that are specific to females, which are similar to the early stages of dilated cardiomyopathy (DCM) (Fig. S1A). In contrast, male mice did not exhibit these changes; instead, they displayed a potential increase in ventricular stiffness (Fig.S1B). These results suggest a sex-specific mechanism involved in the progression of cardiac disease following the loss of A1BG.

### Persistence of Early-Onset cardiac phenotypes in A1bg^CM/CM^ female mice

To determine whether the cardiac phenotypes observed in A1bg^CM/CM^ female mice at 4–6 weeks are resolved or lead to dilated cardiomyopathy, we extended our analyses of females to 6-month-old mice (Fig S1C-D). As previously reported [42, 43], we identified an increase in left ventricular (LV) mass and volume over time; however, no further changes in cardiac function or structure were noted in female A1bg^CM/CM^ hearts compared to control female hearts (Fig. 1G-O). The findings indicate that the loss of A1BG results in an early-onset condition that does not improve over time. Furthermore, the absence of significant remodeling suggests that the heart is not undergoing notable secondary compensatory mechanisms, such as fibrosis or increased hypertrophy, which might otherwise alter the phenotype. Consequently, the early-onset cardiac phenotype observed in A1bg^CM/CM^ female hearts remains persistent, indicating a non-degenerative condition with little to no secondary compensatory remodeling.

### A1BG in female hearts, but not male hearts, regulates the expression of genes related to cardiac metabolism and DCM

**Fig. 2:**
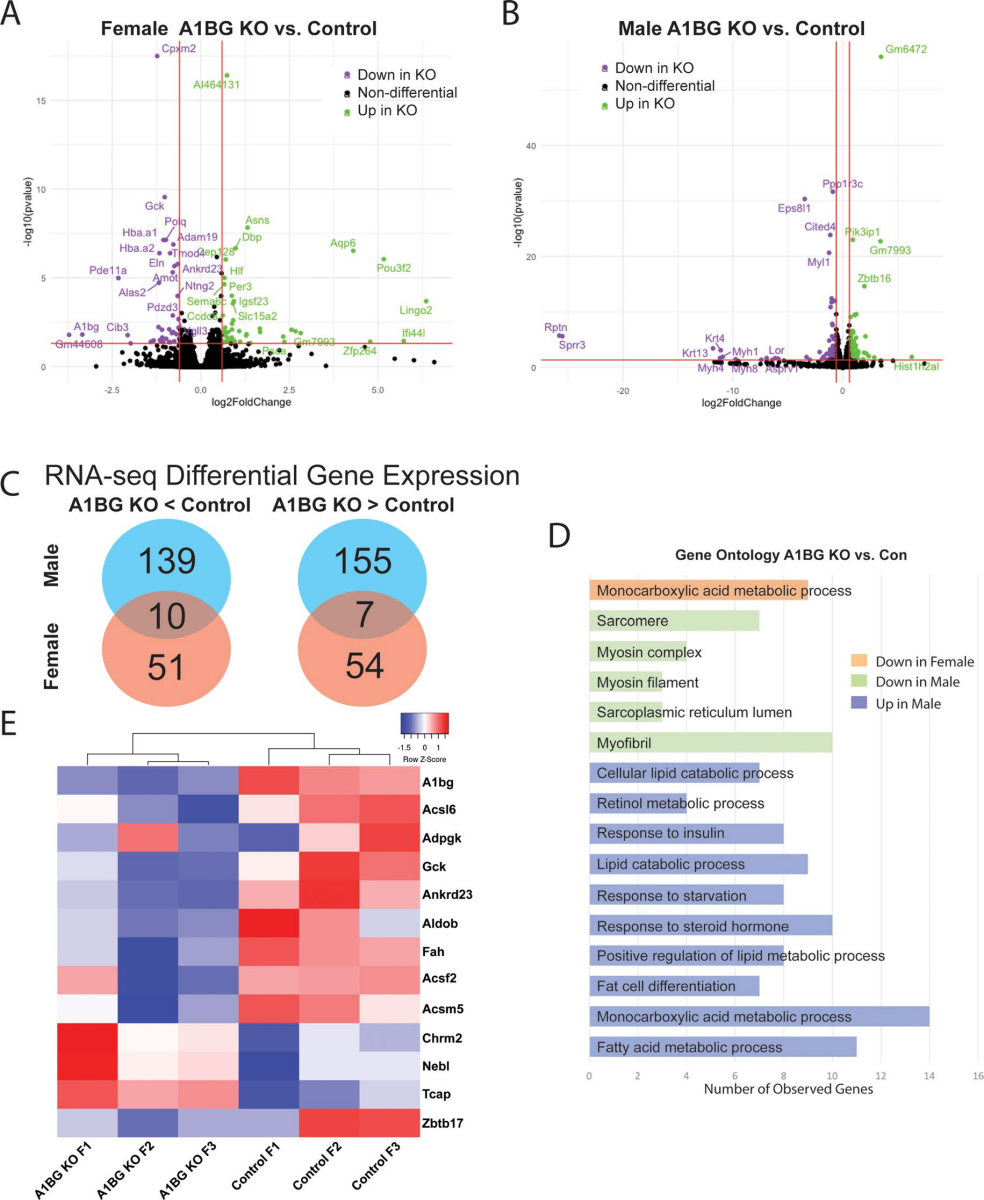
Female hearts, but male hearts, regulate gene expression related to cardiac metabolism and DCM. Volcano plots of differentially expressed genes in (**A**) female KO vs. female control group and (**B**) male KO vs. male control group. Cutoffs used were adjusted p-value < 0.05, and log2 fold change ≥ 0.5 (**C**) Venn diagram depicting genes downregulated (left) and upregulated (right) in A1bg^CM/CM^ (A1BG KO) mice in both males and females. Overlap indicates genes similarly regulated in both sexes in response to A1bg^CM/CM^ (A1BG KO) (**D**) Pathway analysis of differentially expressed genes in response to A1bg^CM/CM^ (A1BG KO). (**E**) Heat map of selected dysregulated genes in female A1bg^CM/CM^ (A1BG KO) and control mice

To understand the molecular underpinnings of A1BG sex-differential effects, transcriptional profiling via RNA sequencing (RNA-seq) was performed on wild-type and A1bg^CM/CM^ male and female hearts at four weeks of age (Fig. 2). This analysis in female hearts identified 122 differentially expressed genes (DEGs; adjusted P-value ≤ 0.05 and log2 fold change ≥ ± 1), of which 61 genes were significantly upregulated, and 61 genes were significantly downregulated compared with controls (Fig. 2A). Though male hearts do not appear to require A1BG, we find that analysis in male hearts identified 311 differentially expressed genes, of which 162 genes were significantly upregulated, and 149 genes were significantly downregulated in A1bg^CM/CM^ male hearts compared with controls (Fig. 2B). Consistent with phenotypic analysis, we find very few genes that are co-regulated in male and female A1bg^CM/CM^ hearts: 10/200 downregulated, and 7/216 were upregulated (Fig. 2C). Therefore, supporting a sex-specific requirement for A1BG in CMs.

Pathway analyses of control versus A1bg^CM/CM^ female hearts revealed a potential role for A1BG in Acetyl-CoA and glucose-6-phosphate metabolism, i.e., “monocarboxylic acids metabolic process” (Fig. 2D). Genes dysregulated in A1bg^CM/CM^ female hearts include Acsl6, Adpgk, Gck, Ankrd23, Aldob, Fah, Acsf2, and Acsm5. None of these genes was dysregulated in male A1bg^CM/CM^ hearts (Fig. 2E). These findings are significant because both Acetyl-CoA and glucose-6-phosphate metabolism are linked to DCM [44–51]. In addition to these genes, we identified 4 genes that were downregulated in the A1bg^CM/CM^ heart, contributing to DCM: Chrm2, Nebl, Tcap, and Zbtb17. Overall, these findings imply a role for A1BG in female hearts for acetyl-CoA and glucose-6-phosphate metabolism, pointing to a critical function of A1BG in preventing female DCM.

### A1BG is required in females to form the cardiac intercalated disc

DCM is a condition characterized by the enlargement and weakened contraction of the left ventricle or both ventricles. This condition is often linked to changes in the connections between heart muscle cells [21–23]. Intercalated discs mediate CM cell-cell connections [52–56]. Mutations in genes related to the intercalated discs in the heart, such as desmoplakin, plakophilin-2, and plakoglobin, which are involved in the formation and function of desmosomes, can cause DCM [52, 54, 57, 58].

**Fig. 3:**
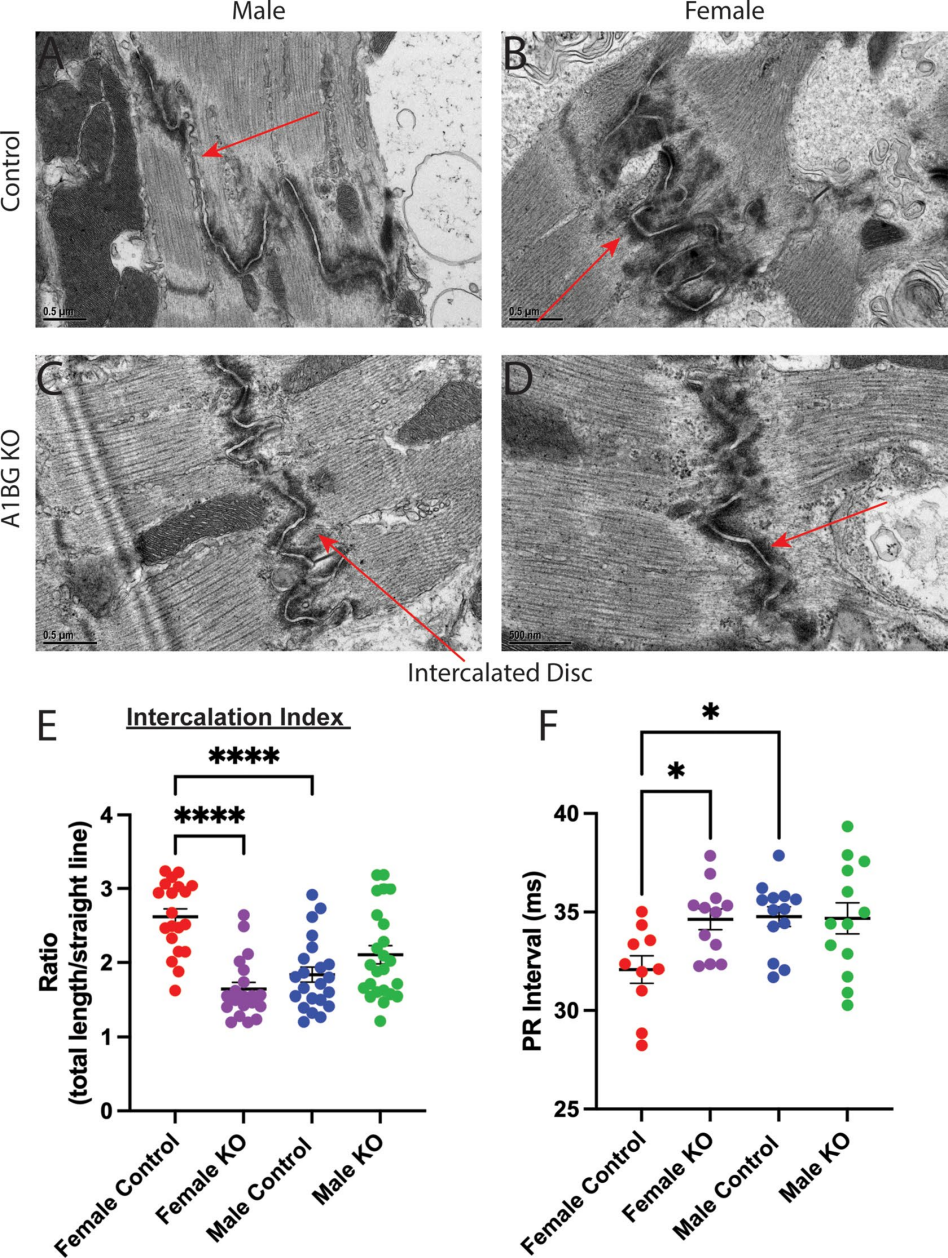
A1BG is required in females for the formation of the cardiac intercalated disc. (**A**-**D**) Transmission electron micrograph images of (**A**) male control, (**B**) female control, (**C**) male A1bg^CM/CM^ (A1BG KO), and female A1bg^CM/CM^ (A1BG KO) (**D**) hearts taken at 50000x magnification. (**E**) Quantification of differences in cardiomyocyte intercalation, taken as a ratio of the total length of cell border divided by straight line length (*n* = 2 hearts per genotype with > 10 intercalated discs per heart analyzed. (**F**) PR interval duration in male and female A1bg^CM/CM^ (A1BG KO, control) and Tnnt2-Cre; A1bg^CM/CM^ (A1BG KO) mice (*n* = 14 mice per genotype). Significance was determined by ANOVA, followed by Tukey’s test. * Indicates *p* < 0.05 between indicated groups, **** indicates *p* < 0.0001 between indicated groups

To test the hypothesis that loss of A1BG in female but not male hearts led to alteration of the cardiac intercalated discs, we used transmission electron microscopy to compare intercalated discs in A1bg^CM/CM^ mice and control mice to determine whether A1BG expression affects intercalated disc structure (Fig. 3A-D). High-resolution (50000x) images uncovered that female A1bg^CM/CM^ intercalated disc morphology was altered compared to control females (Fig. 3A-D).

To quantitatively evaluate the shape of intercalated discs, we measured the ratio of the total length of intercalation to the straight-line length of the cell boundary, as detailed in [29] (Fig. S2A). This allowed us to identify discs with higher values, indicating greater intercalation and, as a result, a larger surface area available for cell adhesion and ion transport. Our analysis showed that female mice had more intercalation than male control mice (Fig. 3E). Female A1bg^CM/CM^ had significantly less intercalation than female controls. In fact, the level of intercalation in female A1bg^CM/CM^ was initially similar to that of male mice. Conversely, male A1bg^CM/CM^ did not differ in intercalated disc shape relative to controls (Fig. 3E). These findings suggest that there are inherent sex differences in cardiac intercalated disc structure and highlight a sex-differential requirement for the role of A1BG in forming intercalated discs.

### A1BG leads to alterations in female cardiac electrophysiology

DCM is associated with alterations in the heart’s electrical properties and conduction pathways [21–23, 59, 60]. Intercalated discs are crucial in coordinating the heart’s contractions by facilitating mechanical and electrical connections between CMs [52–55]. Disruptions in the structure or function of these intercalated discs can significantly affect the heart’s electrical properties, leading to impaired cardiac function [55, 59, 61–64].

Given the intercalation phenotype in female A1BG^CM/CM^ mice, we investigated the electrophysiological consequences of A1BG in both sexes. A1BG is more highly expressed in cardiac atria [33]; therefore, we expected to observe alterations in the electrocardiogram (EKG) PR interval. The PR interval is the time from atrial to ventricular depolarization, indicating the time for electrical impulses to be transmitted through the atria to the AV node (Fig. 3F).

Sex differences exist in human atrial conduction; females have a shorter PR interval than males [65]. This difference was conserved in adult mice, as shown in this study and others (Fig. 3F [38, 66–68]). Further indictive of DCM, the A1BG^CM/CM^ female mice had significantly longer PR intervals than female control mice, indicating a longer time needed for atrial depolarization (Fig. 3F). The female A1BG^CM/CM^ mouse PR interval was similar to the male baseline PR interval (Fig. 3F). As expected, the PR interval was inversely correlated with CM intercalation, with greater intercalation corresponding to shorter PR, affirming the sex-differential role for A1BG in the heart.

### A1BG in females and males interacts with a distinct set of cardiac proteins

There have been limited studies on the function of A1BG. It has been found that the loss of A1BG causes defects in cardiac function that resemble DCM in females but not in males. This observation does not clarify the function(s) of A1BG or explain why there is a different requirement for A1BG based on sex. Additionally, it has been reported that A1BG is one of the most differentially expressed cardiac proteins between males and females in mice at E9.5 and in adults, with higher expression in females than in males [69].

**Fig. 4:**
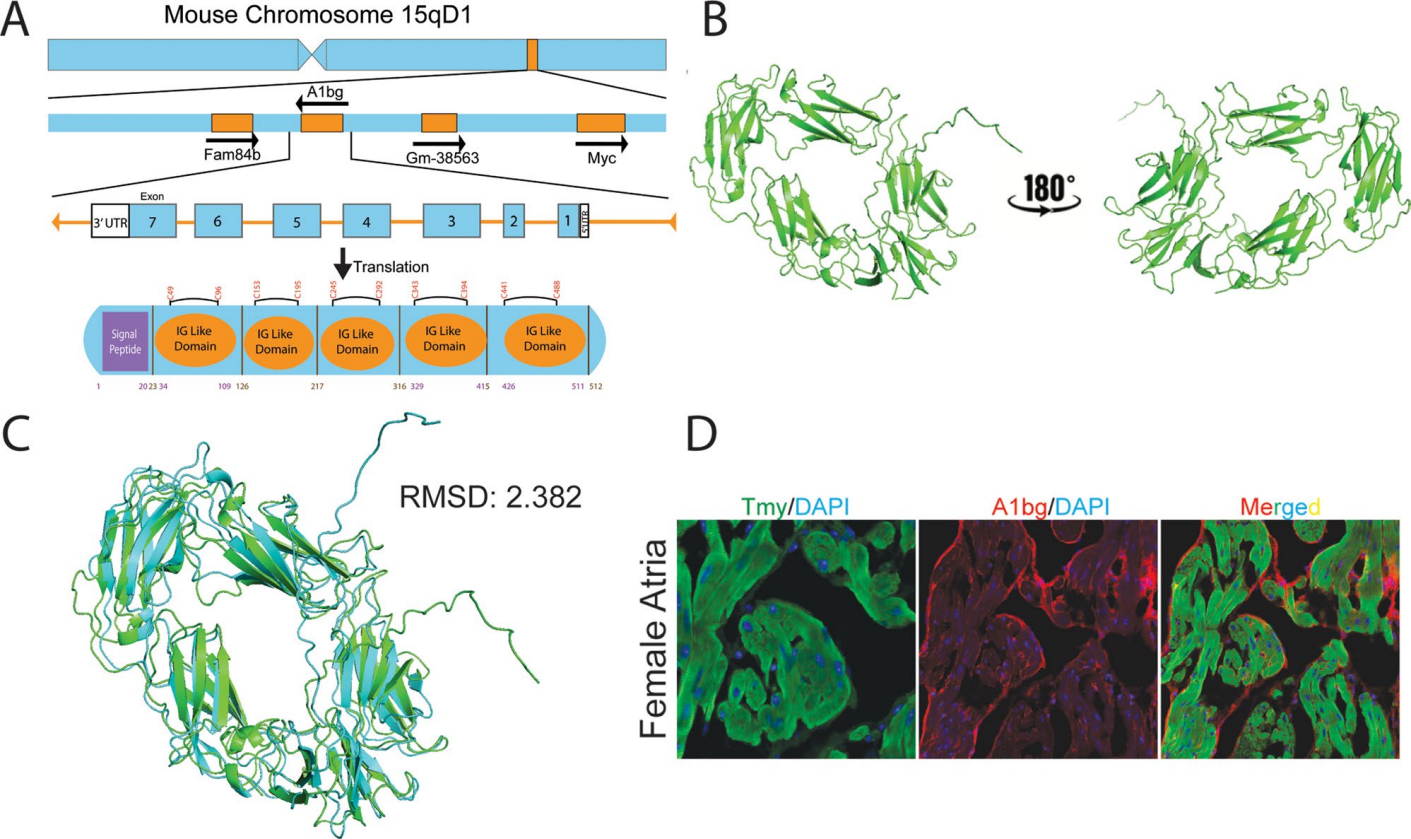
A1BG is an extracellular matrix protein. (**A**) Schematic of A1BG genomic locus, mRNA transcript, and protein with key domains indicated. Amino acid numbers are noted below, and disulfide bonds are above the diagram. (**B**) AlphaFold3 structure prediction of A1BG. (**C**) AlphaFold 3 prediction of human A1BG (blue) aligned with mouse A1bg (green) with root mean square deviation indicated (RMSD) (**D**) Immunohistochemistry staining of female atria with antibodies for tropomyosin (TMY, green), A1BG (red), and DAPI (blue)

To better understand why females specifically require A1BG, we conducted a predicted structural analysis of mouse and human A1BG using Alphafold [70]. Our analysis suggests that the first two exons of the mouse and human A1BG transcript are predicted to encode a signal peptide, and the following five exons encode repeating IgG-like domains (Fig. 4A-C). The structural prediction of mouse and human A1BG suggests that the protein is secreted or associated with the outer cell membrane. We tested this hypothesis by co-immunostaining adult heart tissue in mice with an A1BG and CM (tropomyosin) antibody (Fig. 4D). Our results demonstrate that A1BG is associated with the outer surface of atrial CMs.

**Fig. 5:**
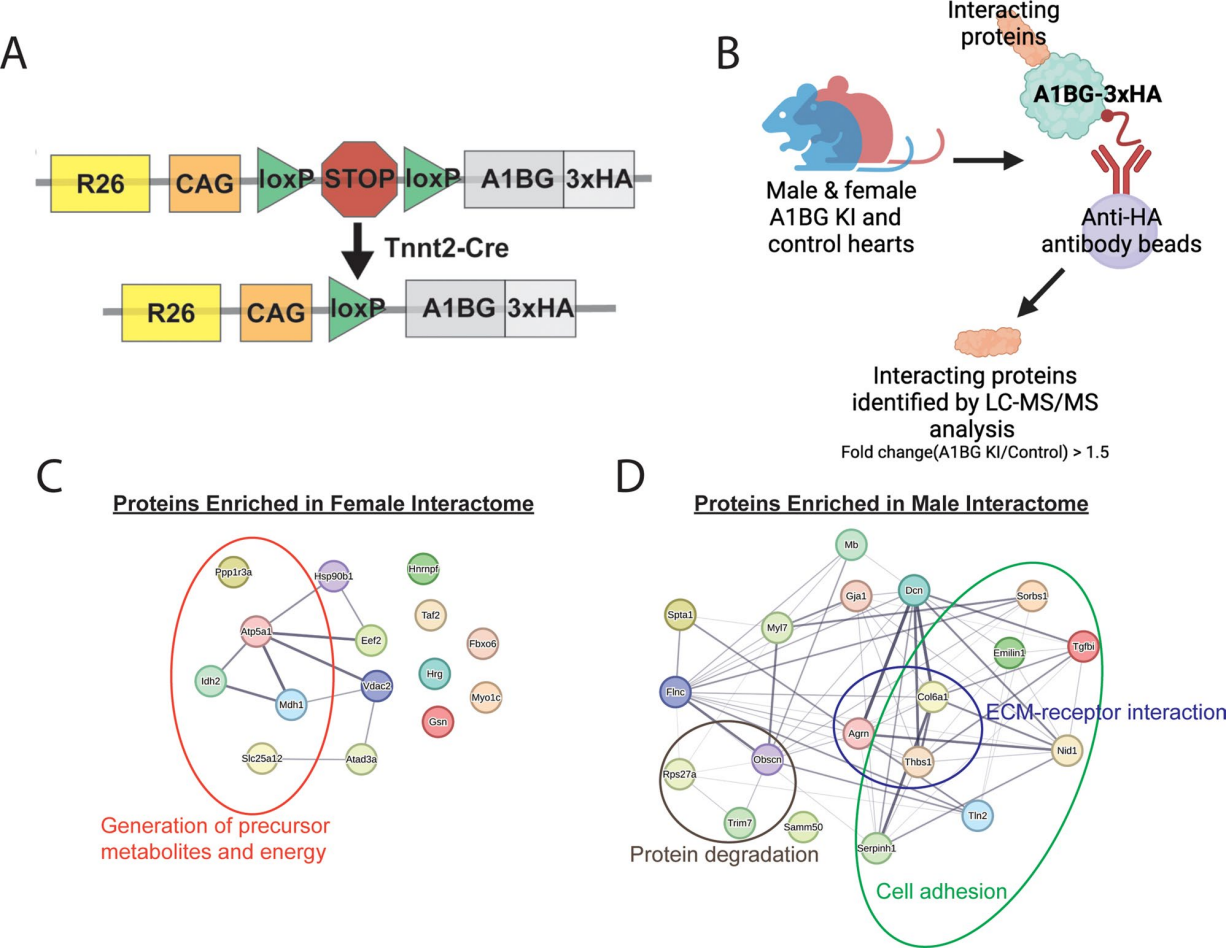
Female and male A1BG cardiomyocyte interactome. (**A**) Schematic of A1BG Rosa26 genomic locus for generation of CM-A1BG^3XHA^ mice. (**B**) Schematic depicting immuno-purification of A1BG in male and female mouse hearts to identify the A1BG interactome by mass spectrometry. Cutoffs were Log fold change > 1.5 A1BG IP vs. non-HA control) (**C**) Proteins enriched in the (**C**) female and (**D**) male A1BG interactome with associated gene ontology circled

The observation that A1BG is a CM cell surface protein, which is required for the proper formation of intercalated discs and cardiac conduction in females but not males, as well as the structural prediction of A1BG, has led us to characterize cardiac A1BG interactomes in females and males. Researchers have not found a highly specific, high-affinity antibody against mouse A1BG that can function in immune-affinity purification. To address these issues, we generated an inducible A1BG allele by knocking an epitope-tagged version of A1BG (A1BG-3xHA) flanked by flox-stop-flox cassettes into the ROSA26 locus to create A1BG^3XHA^ (Fig. 5A). To induce expression of A1BG^3XHA^ in CMs we crossed the A1BG^3XHA^ to cTnt-Cre mice, CM-A1BG^3XHA^ (Fig. 5A). F1 and F2 heterozygous and homozygous CM- A1BG^3XHA^ mice were viable and fertile and had no observable phenotypic abnormalities and expression in the F2 was confirmed by immunoblot with anti-HA antibodies (Fig. 5A, Fig. S2B).

To deduce the function of A1BG in cardiac tissue and to further explore the sex difference requirements for A1BG, we defined the A1BG endogenous cardiac interactome by performing mass spectrometry (MS) analysis of immuno-affinity purified (IP-MS/MS) female A1BG^3HA^ CM complexes [40, 71, 72] (Fig. 4B). The complexes (*N* = 3) were obtained under physiological conditions from CMs derived from the hearts of female and male CM-A1BG^3XHA^ mice at 4 weeks of age (Fig. 4B) in the presence of RNAse and DNAse. Results demonstrate that we could recover A1BG^3XHA^ at 73%, near the theoretical maximum with a trypsin digest (Fig. S3).

The analysis of CM-A1BG^3XHA^ complexes utilized an unbiased gene ontology-based bioinformatics classification to scrutinize the functions of proteins linked with A1BG. Functional network analyses clearly showed that A1BG interacts with a group of 15 proteins enriched in females and 19 enriched in males (Fig. 5C, D). Upon conducting gene ontology analysis, it was apparent that the female interactome is enriched with proteins involved in generating precursor metabolites and energy, while the male interactome is enriched in extracellular matrix (ECM)-receptor interaction and cell adhesion proteins (Fig. 5C, D; Table 1). Among the 15 proteins found to be enriched in females, 7 have not undergone a study in the context of the heart, while the remaining 8 have been linked to cardiac disease, including DCM (Table 1). None of the female A1BG interacting proteins were identified in the male A1BG cardiac interactome (Fig. 5C, D; Tables 1 and 2). Consistent with our 6-week echocardiographic analysis (Fig. 1, S1), the male A1BG cardiac interactome comprises proteins involved in protein degradation. These proteins were absent in the female cardiac interactome (Fig. 5C, D; Tables 1 and 2). Thus, the specific set of interacting proteins differed significantly from that in females. Female interactomes are enriched in proteins related to energy metabolism and are associated with DCM pathologies. Our findings suggest a sex-specific requirement for A1BG in cardiac health and imply that A1BG interactions may underlie the sex-specific requirements for A1BG in cardiac function.

**Table 1:** Proteins enriched in female A1BG IP

Protein name	Description	Connection to cardiac physiology
Slc25a12	Calcium-binding mitochondrial carrier protein Aralar1	NA
Atp5f1a	ATP synthase subunit alpha, mitochondrial	NA
Myo1c	Unconventional myosin-Ic	NA
Eef2	Elongation factor 2	Pathological hypertrophy (Varma et al., 2023[76])
Idh2	Isocitrate dehydrogenase [NADP], mitochondrial	Cardiac hypertrophy (Wu et al., 2022[77], Ku et al., 2015[78])
Fbxo6	F-box only protein 6	NA
Taf2	Transcription initiation factor TFIID subunit 2	NA
Ppp1r3a	Protein phosphatase 1 regulatory subunit 3 A	Atrial fibrillation (Alzina et al., 2019[79]), Heart failure Cordero et al., 2019[80])
Gsn	Gelsolin	Myocardial infarction (Li et al., 2009[81]), Atrial fibrillation (Schrickel et al., 2009[82])
Atad3	ATPase family AAA domain-containing protein 3	Perinatal cardiomyopathy (Frazier et al., 2021[83])
Hrg	Histidine-rich glycoprotein	NA
Hsp90b1	Endoplasmin	Kawasaki disease (Mingguo et al., 2020[84])
Hnrnpf	Heterogeneous nuclear ribonucleoprotein F	NA
Mdh1	Malate dehydrogenase, cytoplasmic	Acute myocardial infarction (Pan et al., 2020[85])
Vdac2	Voltage-dependent anion-selective channel protein 2	Dilated cardiomyopathy (Shankar et al., 2021[86])

**Table 2:** Proteins enriched in male A1BG IP

Protein name	Description	Connection to cardiac physiology
Gja1	Gap junction alpha-1 protein	Arrhythmogenic cardiomyopathy (Palatinus 2023[87])
Nid1	Nidogen-1	NA
Col6a1	Collagen alpha-1(VI) chain	Trisomy 21 congenital heart disease (Davies et al., 1995[88])
Flnc	Cluster of Filamin-C	Hypertrophic & Dilated cardiomyopathy (Verdonscot et al., 2020[89])
Tln2	Talin-2	Atrial septal defect (Teekakirikul et al., 2022[90])
Thbs1	Cluster of Thrombospondin-1	NA
Myl7	Myosin regulatory light chain 2, atrial isoform	NA
Macroh2a1	Core histone macro-H2A.1	NA
Sorbs1	Sorbin and SH3 domain-containing protein 1	NA
Dcn	Decorin	NA
Samm50	Sorting and assembly machinery component 50 homolog	Promotes hypertrophy (Xu et al., 2021[91])
Trim7	E3 ubiquitin-protein ligase TRIM7	NA
Agrn	Agrin	Catecholaminergic polymorphic ventricular tachycardiac (Jaouadi et al., 2022[92])
Mb	Myoglobin	Myoglobinopathy (Olive et al., 2019[93])
Serpinh1	Serpin H1	NA
Emilin1	EMILIN-1	Aortic valve disease (Munjal et al., 2014[94])
Spta1	Spectrin alpha chain, erythrocytic 1	NA
Tgfbi	Transforming growth factor-beta-induced protein ig-h3	Atrial fibrillation (Guan et al., 2022[95])
Obscn	Obscurin	Hypertrophic cardiomyopathy (Wu et al., 2021[96]), Arrhythmogenic right ventricular cardiomyopathy (Chen et al., 2020[97])
rps27a	ribosomal protein 27a	NA

## Discussion

Here, we show that the absence of A1BG in female mice leads to persistent structural remodeling of the left ventricle, including ventricular dilation and wall thinning, consistent with early-stage dilated cardiomyopathy (DCM). Although echocardiographic assessment at 6 months of age reveals preserved systolic function—indicated by unchanged ejection fraction (EF) and fractional shortening (FS)—these structural abnormalities are sustained over time, suggesting a chronic pathological remodeling process rather than a transient or compensatory response. This finding supports the hypothesis that A1BG plays a critical role in maintaining normal ventricular structure in female hearts and may contribute to sex-specific susceptibility to pathological remodeling that can precede overt functional decline.

A1BG influences the electrophysiological properties of the heart in females and not males. Female A1bg^CM/CM^ mice had a significantly longer PR interval on electrocardiograms (EKG), indicating delayed atrial depolarization. Our histological analysis and echocardiogram data showed that female A1BG^CM/CM^ hearts (indicated by LV mass) were smaller than the hearts of female controls. Therefore, the change in the PR interval is not attributed to the size difference between female and male hearts.

Based on our findings on the predicted structure of A1BG, our observation from immunohistochemistry that A1BG is associated with the CM ECM, and the composition of the female interactome, we favor a model by which female CMs require A1BG to establish CM cell-cell contact. This defect, in turn, leads to a dysregulation of genes (e.g., Csl6, Adpgk, Gck, Ankrd23, Aldob, Fah, Acsf2, and Acsm5), suggesting a shift towards a higher dependence on glucose oxidation in female hearts. In conjunction with other pathological changes, this metabolic adaptation likely contributes to the structural and functional remodeling observed in the 6-week-old female heart, typical in DCM.

A key unanswered question is why A1BG is not needed in male hearts. We propose that A1BG’s role in females is to protect the heart from cardiac stress, such as DCM. As estrogen protects against various cardiovascular diseases, including DCM, by influencing cardiac metabolism, gene expression, and structural integrity [14, 28–30, 73, 74], we propose that A1BG acts downstream or in parallel to estrogen signaling. Our observation indicates that female mice with a loss-of-function A1BG gene (A1bg^CM/CM^) show significant cardiac dysfunction and morphological changes consistent with DCM, while their male counterparts do not. This suggests that A1BG may add to the protective role of estrogen. Understanding the relationship between A1BG and estrogen in cardiac function could lead to targeted therapeutic strategies for treating or preventing DCM, particularly in females.

Although A1BG is only 63% conserved between mice and humans, its structure remains remarkably conserved, with a root mean square deviation of a mere 2.382 in predicted structural alignments. This structural alignment suggests that A1BG has a similar function in the two species, accentuating its potential role in therapeutic strategies for cardiac conditions such as DCM. Given the role of A1BG in CMs, targeting the A1BG pathway in female patients could be particularly impactful. Metabolic interventions that address dysregulations in acetyl-CoA and glucose-6-phosphate metabolism may mitigate imbalances associated with DCM [75]. Considering the sex-specific requirements for A1BG, further research into A1BG interactomes in both sexes is imperative to develop new therapeutic biomarkers and targets. Thus, it is essential to use sex-specific approaches in the treatment of cardiac disorders linked to the A1BG pathway.

## Electronic supplementary material

Below is the link to the electronic supplementary material.

Supplementary Material 1

Supplementary Material 2: Echocardiography data (A) Echocardiogram data from 4–6-week-old female control and female A1BG KO mice, and (B) male control and male A1BG KO mice (n = 11 for both). (C) Echocardiogram data from 6-month-old female A1BG KO mice compared to female control mice (n = 7). (D) Change in mean values between 6 weeks and 6 months for female control and female A1BG KO mice (All significance determined by students t test, * indicates p < 0.05) (IVS– interventricular septum thickness, LVID– Left ventricular interior diameter, LVPW– left ventricular posterior wall, EF– ejection fraction, FS- fractional shortening, LV Vol- estimated volume of left ventricle,;s– systole,;d- diastole).

Supplementary Material 3: Quantification of interaction index (A) Diagram depicting quantification of intercalation index as used in Fig. 3E. (B) Western blot against HA-tag in male and female Tnnt2-Cre; R26-A1BG-3HA+/+(CM-A1BG^3XHA^) confirming protein is expressed and at the expected molecular weight.

Supplementary Material 4: A1BG immuno-purification peptide recovery This figure highlights the amino acids for A1BG that were identified in mass spectrometry. 73% of possible A1BG amino acids were identified, which was 76% of the theoretical maximum using a trypsin digest.

## Data Availability

No datasets were generated or analysed during the current study.
