# Supplementary figures and images for: Sex-specific response to A1BG loss results in female dilated cardiomyopathy
Source: Biol Sex Differ. 2025 Apr 23;16:27. doi: 10.1186/s13293-025-00713-8 (PMC12016195; doi:10.1186/s13293-025-00713-8)

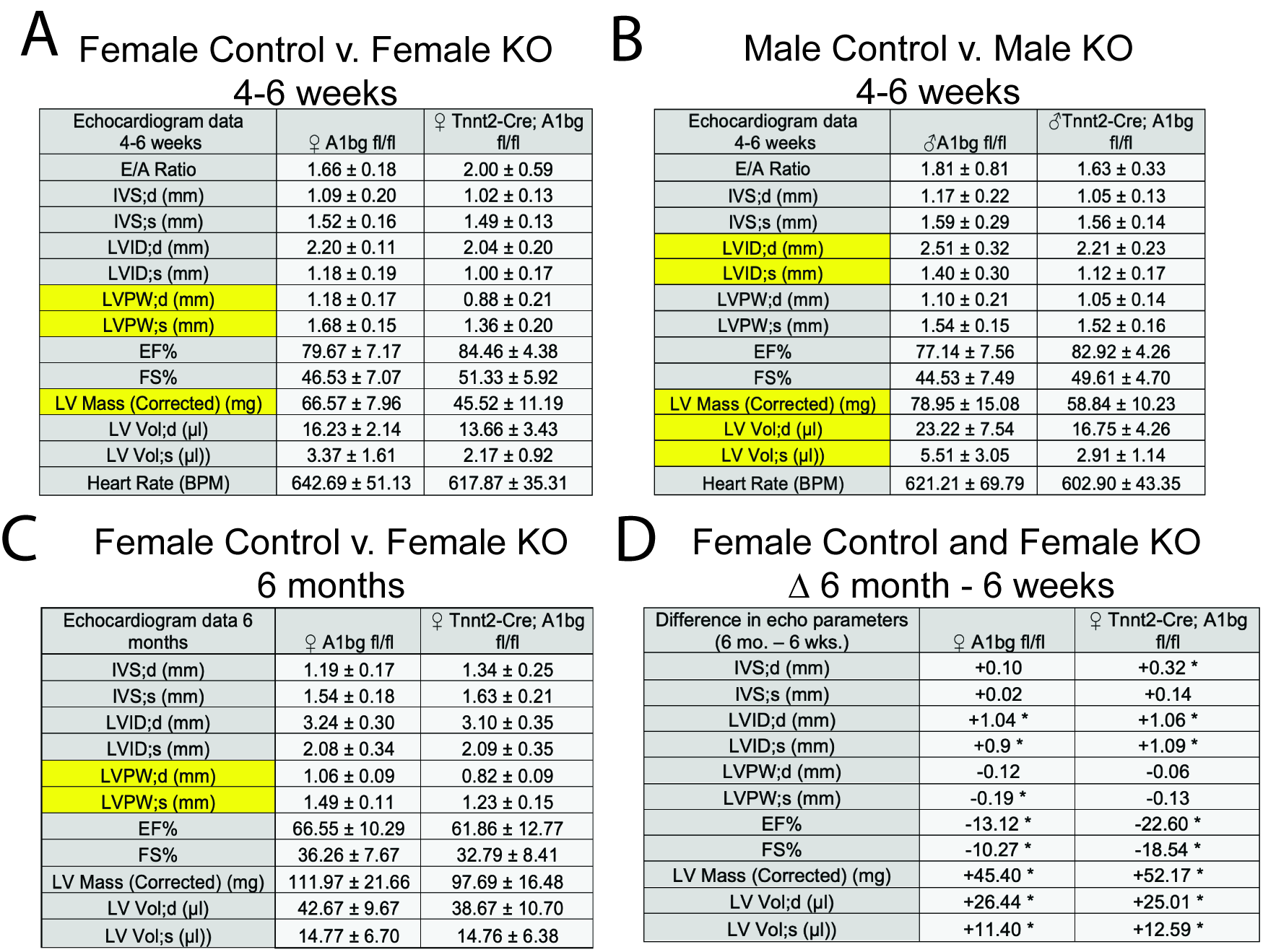

Supplement: Supplementary file 2 — Supplementary Material 2: Echocardiography data (A) Echocardiogram data from 4–6-week-old female control and female A1BG KO mice, and (B) male control and male A1BG KO mice (n = 11 for both). (C) Echocardiogram data from 6-month-old female A1BG KO mice compared to female control mice (n = 7). (D) Change in mean values between 6 weeks and 6 months for female control and female A1BG KO mice (All significance determined by students t test, * indicates p < 0.05) (IVS– interventricular septum thickness, LVID– Left ventricular interior diameter, LVPW– left ventricular posterior wall, EF– ejection fraction, FS- fractional shortening, LV Vol- estimated volume of left ventricle,;s– systole,;d- diastole). [file 13293_2025_713_MOESM2_ESM.tif]

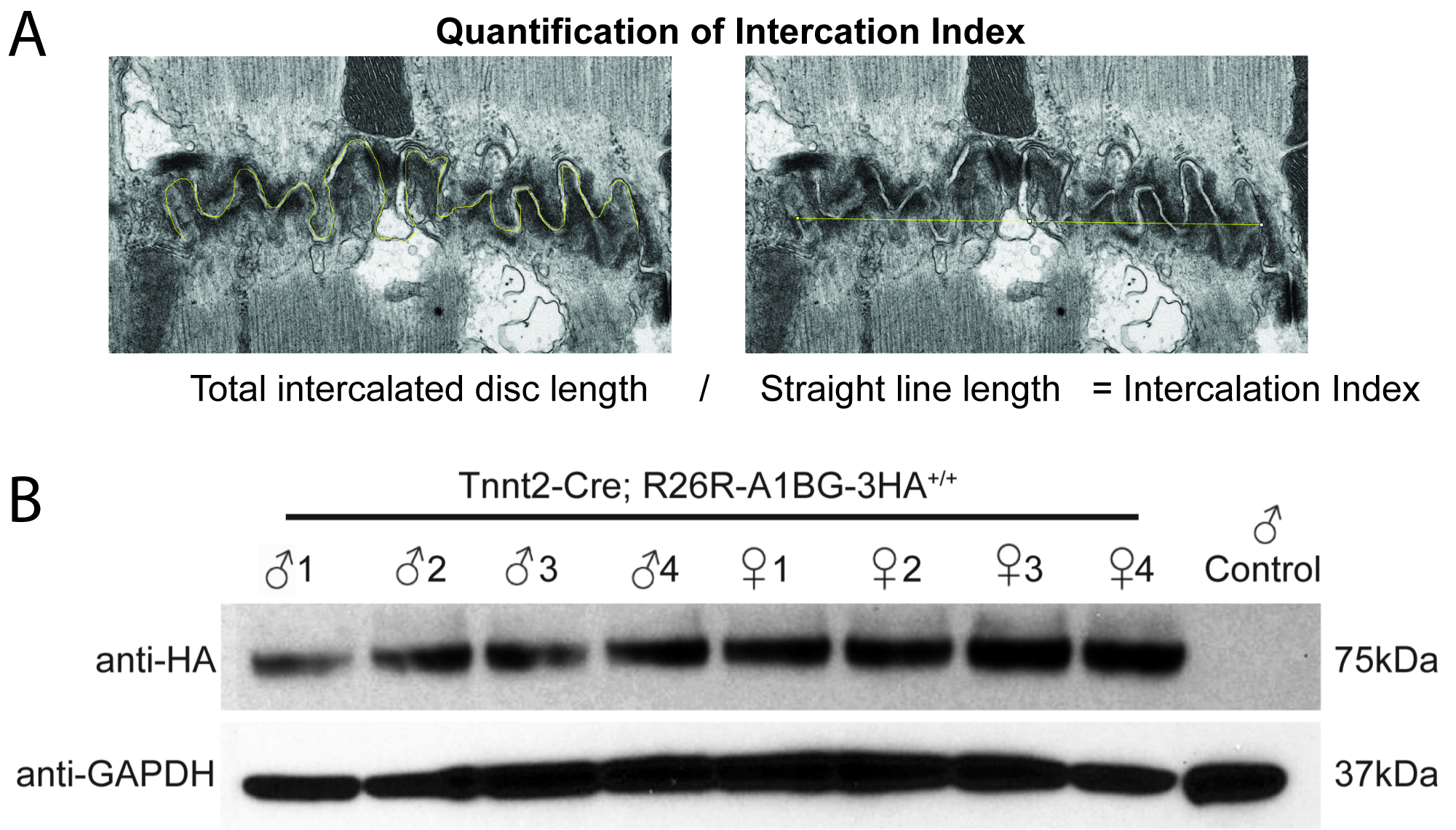

Supplement: Supplementary file 3 — Supplementary Material 3: Quantification of interaction index (A) Diagram depicting quantification of intercalation index as used in Fig. 3E. (B) Western blot against HA-tag in male and female Tnnt2-Cre; R26-A1BG-3HA+/+(CM-A1BG3XHA) confirming protein is expressed and at the expected molecular weight. [file 13293_2025_713_MOESM3_ESM.tif]

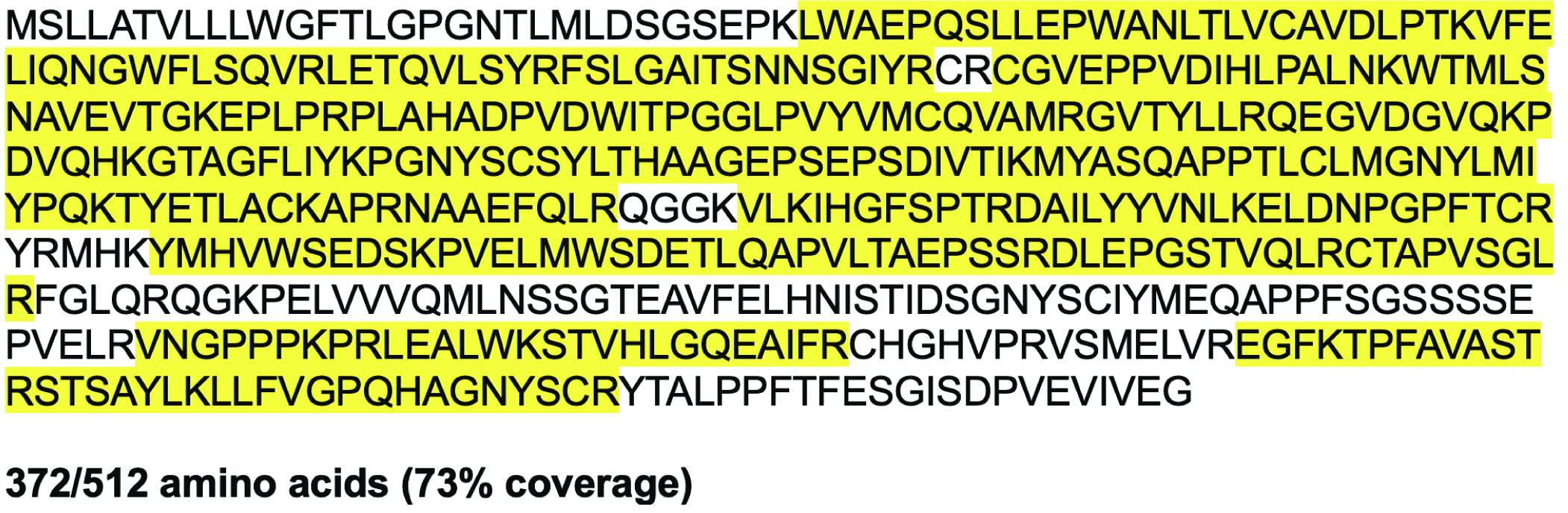

Supplement: Supplementary file 4 — Supplementary Material 4: A1BG immuno-purification peptide recovery This figure highlights the amino acids for A1BG that were identified in mass spectrometry. 73% of possible A1BG amino acids were identified, which was 76% of the theoretical maximum using a trypsin digest. [file 13293_2025_713_MOESM4_ESM.tif]
